# Evaluation of the impact of Tacrolimus-based immunosuppression on Heidelberg liver transplant cohort (HDTACRO): Study protocol for an investigator initiated, non-interventional prospective study

**DOI:** 10.1097/MD.0000000000022180

**Published:** 2020-09-25

**Authors:** Elias Khajeh, Georgios Polychronidis, Ali Ramouz, Parnian Alamdari, Anastasia Lemekhova, Melisa Saracevic, Sadeq Ali-Hasan-Al-Saegh, Omid Ghamarnejad, Ali Majlesara, Sepehr Abbasi Dezfouli, Arash Nickkholgh, Karl Heinz Weiss, Christian Rupp, Arianeb Mehrabi, Markus Mieth

**Affiliations:** a Department of General, Visceral, and Transplantation Surgery, Heidelberg University Hospital; b Department of Gastroenterology and Hepatology, University of Heidelberg; c Department of Internal Medicine, Heidelberg University Hospital, Heidelberg, Germany.

**Keywords:** immunosuppression, liver transplantation, tacrolimus

## Abstract

**Background:**

Tacrolimus-based immunosuppression has resulted in enormous improvements on liver transplantation (LTx) outcomes. However, dose adjustment and medication adherence play a key role in post-transplant treatment success. The aim of the present study is to assess the trough levels and the need for adaptation of therapeutic doses in de novo LTx patients treated with Tacrolimus in the clinical routine, without any intervention to the treatment regimen.

**Methods and analysis:**

This is a pilot, prospective, exploratory, monocentric, non-interventional and non-randomized investigator-initiated study. Prospectively maintained data of 100 patients treated with various oral Tacrolimus-based immunosuppressants (Prograf or Envarsus) will be analyzed. The number of required dose adjustments of Tacrolimus formulations used in clinical routine for achieving the target trough level, Tacrolimus trough level, Tacrolimus dosing, concentration/dose ratio, routine laboratory tests, efficacy data (incl. survival, acute rejection, re-transplantation), patients therapy adherence, and infections requiring the need to reduce individual immunosuppressant dosing will be evaluated for each patient.

**Result:**

This study will evaluate the trough levels and the need for adaptation of therapeutic doses in de novo LTx patients treated with Tacrolimus in the clinical routine, without any intervention to the treatment regimen.

**Conclusion:**

The HDTACRO study will be the first study to systematically and prospectively evaluate various oral Tacrolimus-based immunosuppressants in de novo liver transplanted patients. If a difference between the therapy-subgroups is evident at the end of the trial, a randomized control trial will eventually be designed. Registration number: ClinicalTrials.gov: NCT04444817.

## 1 Introduction

Modern immunosuppression is characterized by a combination of different immunosuppressants.^[1,2]^ Immunosuppression based on low-dose calcineurin inhibitors (CNI), with comparatively low CNI target levels, have shown satisfying outcomes in preventing chronic graft rejection.^[1–3]^ Low-dose CNI immunosuppression is of great importance considering the often poor condition of the transplant candidates in the course of the model for end-stage liver disease (MELD) score-based organ allocation system. Despite all efforts to optimize the treatment regimen after liver transplantation (LTx) from deceased donors, the amount of postoperative medication remains high, with CNIs being the main component of immunosuppressive treatment. Tacrolimus is known as the main substance, which can be administered in combination with steroids and possibly mycophenolic acid. Tacrolimus requires an individual dose titration due to its narrow therapeutic index, to achieve a satisfactory balance between maximizing efficacy and minimizing dose-related toxicity.^[4]^ The pharmacokinetic profile of Tacrolimus is characterized by a high degree of inter- and intraindividual variability. Although it is rapidly absorbed, the bioavailability of Tacrolimus in the twice-daily capsule formulation is low and variable, ranging from 17% to 23%.^[5]^ This could be due to poor water solubility, extensive first pass metabolism, p-glycoprotein-mediated efflux and the ingestion of food.^[6]^ Tacrolimus twice-daily capsules are also associated with a unique high peak following dosing, which may be associated with increased toxicity.^[7,8]^ Furthermore, graft recipients ought to receive very demanding medication regimen for a long time, which might be associated with decreased patients adherence.,^[9–11]^ which can lead to rejection and possibly graft loss.^[12,13]^

The development of once-daily Tacrolimus formulations has already been shown to increase patients adherence.^[14,15]^ Although, it has slightly influenced the Tacrolimus pharmacokinetic profile, while the dissolving form still remains intact.^[14,15]^ The pharmacokinetic profile of once-daily Tacrolimus is characterized by flatter kinetics (i.e., less fluctuation and swing) compared to a twice-daily regimen. Thus, once-daily Tacrolimus can also lead to reduced incidence and/or intensity of drug toxicity-related adverse events, by providing a more balanced concentration-time consistency over 24 hours. Since the development of LCP-Tacrolimus once-daily tablets, using the MeltDose technology, clinical data have shown lower peak and reduced peak-to-trough fluctuations.,^[16,17]^ as well as an improved clinical safety profile.^[18]^ Furthermore, trials with stable kidney or liver transplant recipients who were switched from twice-daily Tacrolimus capsules to once-daily LCP-Tacrolimus tablets showed a similar area under the concentration–time profile (AUC24) with reduced doses of LCP-Tacrolimus.^[19–21]^

The aim of the present study is to assess the trough levels and the need for adaptation of therapeutic doses in de novo LTx patients treated with Tacrolimus in the clinical routine, without any intervention to the treatment regimen.

## 2 Methods

### 2.1 Study settings

This is a pilot, prospective, exploratory, monocentric, non-interventional, and non-randomized investigator-initiated study to assess practicability and efficacy of Tacrolimus used in de novo liver transplant recipients. The study protocol has been registered at ClinicalTrials.gov (registration number: NCT04444817). Prospectively maintained data of 100 patients treated with various oral Tacrolimus-based immunosuppressants (Prograf or Envarsus) will be analyzed. The study is expected to last for at least 3 years.

### 2.2 Course of the study

As shown in Table 3, the treatment and patient care will follow standard clinical practice for liver transplant patients at the Department of General, Visceral and Transplantation Surgery, University Hospital Heidelberg. The individual treatment decision will be taken independently from the participation in this non-interventional study. For all patients a total of 8 visits, as part of clinical routine, is planned from visit 1/day 0 until visit 8/day 180 (end of study) (Table 3). All data until visit 5 (day 7) will retrospectively be observed via clinical IT-System i.s.h.med of the University Hospital Heidelberg. On the fifths patient visit (post-operative day 7), the informed consent will be obtained from all participants. From this time point on, all data will be observed prospectively and documented (Table 3). Similar to the clinical routine, as soon as a patient is able to swallow and has a sufficient gastrointestinal activity, Tacrolimus-based immunosuppression using Prograf, or Envarsus will be started. Furthermore, CNI-based immunosuppression will be used (initial dose based on the patients body weight) with the goal of trough levels of 3 to 7 ng/ml within the first seven days after LTx, depending on immune status and indication for transplantation. Further trough levels will be determined based on factors such as patients history and indication for LTx. To minimize renal and infectious complications, a “bottom-up” dosing procedure (as a concept of delayed CNI-based immunosuppression) will be routinely used.

### 2.3 Patient selection

Inclusion criteria has been determined according to the standard clinical practice for liver transplant patients. Inclusion and exclusion criteria have been summarized in Table 1. The eligibility will be determined based on the informed consent status, age, present condition, and planned surgery.

**Table 1:**
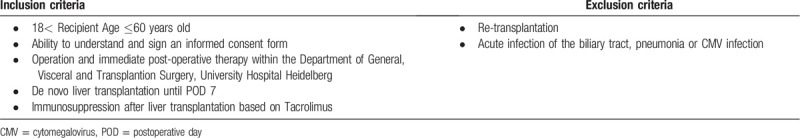
Inclusion and exclusion criteria of the HDTACRO study.

### 2.4 Outcome measures

#### 2.4.1 Primary endpoint

The primary endpoint of the present study is to analyze the number of required dose adjustments of various Tacrolimus formulations, as used in clinical routine, until the individual target trough level in de novo liver transplant patients is achieved.

#### 2.4.2 Secondary endpoints

As presented in Table 2, demographics and clinical characteristics of donors and recipients will be reported. Tacrolimus related information including: Tacrolimus trough level, Tacrolimus dosing, concentration/dose ratio, mean cumulative dose for cost analysis, will be recorded during patient visits. Furthermore, routine laboratory tests will be assessed and reported for each patient. Finally, efficacy data (incl. survival, acute rejection, re-transplantation), patients therapy adherence, and infections requiring the need to reduce individual Immunosuppressant dosing will be evaluated for each patient. Afterwards, the number of patients who require a change in immunosuppressive therapy, the stability of Tacrolimus trough levels and effectiveness of delayed CNI-based immunosuppression with Tacrolimus will be determined and the renal and transplant function under delayed CNI-based immunosuppression with Tacrolimus will be assessed.

**Table 2:**
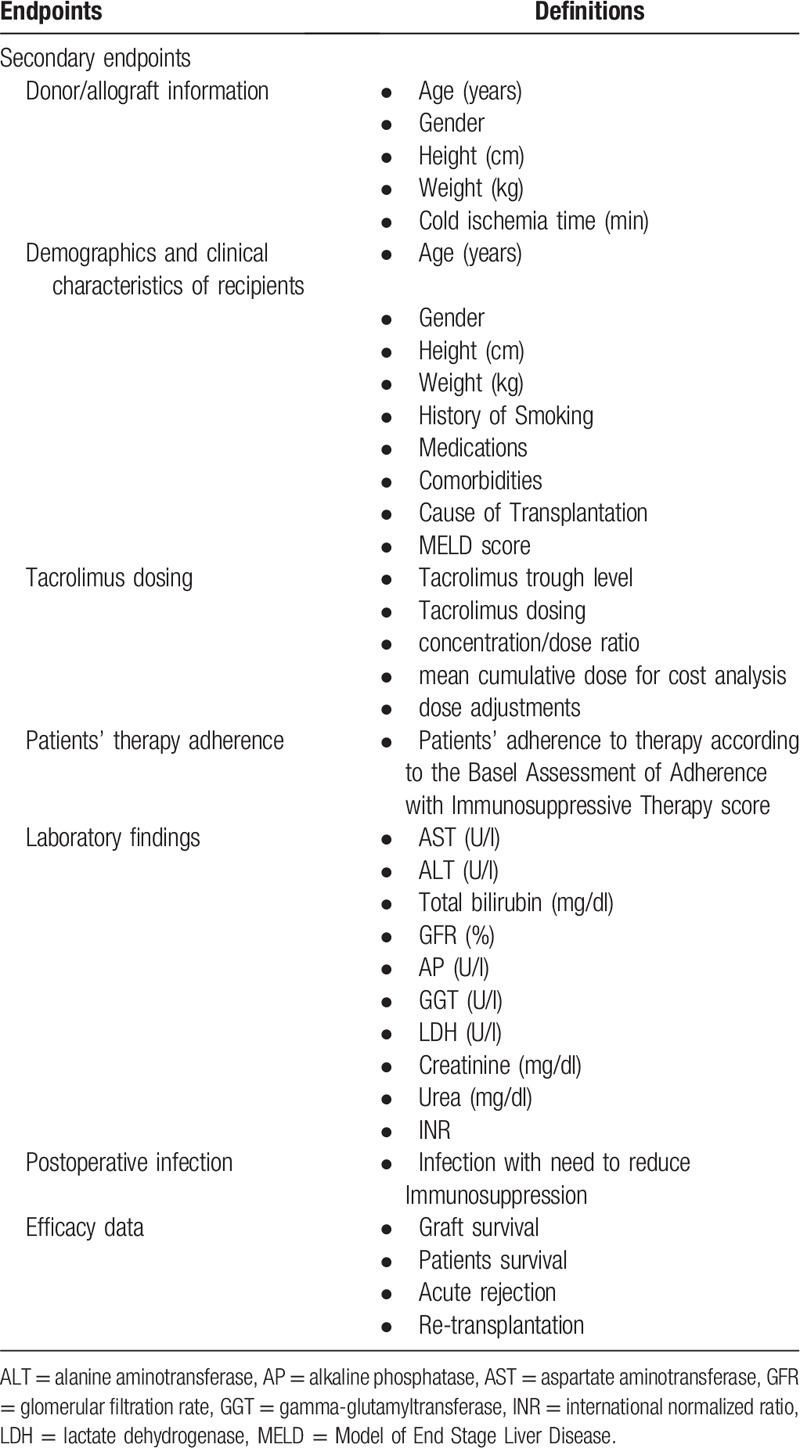
Secondary endpoints of the HDRACRO study.

**Table 3:**
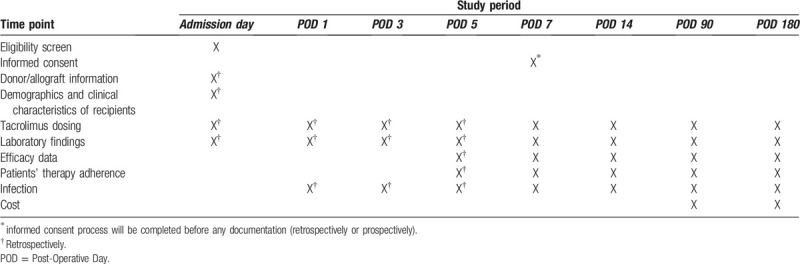
HDTACRO study design according to the SPIRIT checklist.

### 2.5 Patient and public involvement

The patients and public were not involved in the planning of this study.

### 2.6 Modification of the protocol

Protocol amendments will be considered by the principal investigator. All potential amendments will be submitted to the independent Ethics Committee of the University of Heidelberg for approval. No patients will be recruited until the modifications are accepted.

### 2.7 Methods for minimizing bias

To avoid selection bias and heterogeneity, all patients admitted to University Hospital Heidelberg for LTx will be screened for eligibility. Every patient who meets the eligibility criteria will be informed of the study seven days after transplantation, and will be included if he/she gives consent to participate. Data will only be analyzed after all data have been collected. Any financial relationship and any conflict of interest will also be declared.

### 2.8 Data management

Medical history and adverse drug reactions will be coded using the MedDRA dictionary. Medications will be coded using the WHO Drug dictionary and Anatomical Therapeutic Chemical classification. All collected data will be documented in a paper-based case report form (CRF). Data entry into CRFs at the site will be accomplished by qualified site personnel only. Entered data will be reviewed by manual reviews. Front-end edit checks will be used in addition to the manual data review to check for discrepancies and to ensure consistency and completeness of the data. After cleaning of data, a review meeting will be held to determine the occurrence of any protocol violation and to define the subject populations for the analysis. Once the database has been declared to be complete and accurate, it will be locked and the planned statistical analysis will be performed. Only authorized and well-documented updates to the study data are possible after database lock.

### 2.9 Statistical design and analysis

#### 2.9.1 Sample size

Since no confirmatory approach will be followed in this non-interventional study, no formal sample size estimation was carried out. For practical reasons, the number of included subjects was set to 100, which are deemed to be sufficient to provide stable estimates of the primary variable.

#### 2.9.2 Statistical analysis

Dichotomous data will be presented as frequencies. Continuous data will be presented as means ± standard deviation, and in case of skewed distributions medians and ranges. To evaluate the difference between various Tacrolimus types, subgroup analysis will be performed. Accordingly, dichotomous data will be analyzed using Pearsons Chi-Squared test and continues data using repeated measure analysis of variance (ANOVA) model. A two-sided *P* value <.05 will be considered statistically significant in all analyses.

Since the amount of dose adjustments has only been partially described, the total number will be documented for each patient and evaluated as a dichotomous variable for each visit as well as a continuous variable for the time-frame between visits. Recent literature suggests that a stabilization of trough levels does not require longer than a period of 30 days for both de novo immunosuppression and conversion.^[22,23]^

### 2.10 Ethics and dissemination

The independent Ethics Committee of the University of Heidelberg has approved the protocol of present study (registration number: S-630/18). This study was designed in accordance with the Declaration of Helsinki, version 2013. Participation will be voluntary and consent may be withdrawn at any time, without explanation and with no impact on further medical care. The execution of the trial is carried out in the Department of General, Visceral and Transplantation Surgery of the University Hospital Heidelberg. According to paragraph 4 Abs. 23 AMG the medical treatment of the patients will be undertaken based on their diagnosis and clinical treatment and not based on this protocol. The results of this study will be published in a peer-reviewed journal, and will also be presented at medical meetings.

## 3 Discussion

LTx is widely used as a life-saving operation for patients with end-stage liver disease. LTx is also a standard therapy for some inherited metabolic disorders like familial hypercholesterolemia and malignancies with liver involvement such as hepatocellular carcinoma and hepatoblastoma. Recent progress in immunosuppression and medical management, procurement and preservation, and technical accomplishments have continually improved patient survival.^[23,24]^ Orthotopic LTx has shown a noticeable survival rate (83% for 1 year and 75% for 5 years) with remarkable improvements over the past 3 decades, owing to new agents and changed regimens of post-transplant immunosuppression.^[23,24]^ Although long-term post-transplant immunosuppression reduces rejection in LTx recipients, it increases the risk of infections, malignancies, and specific adverse side-effects unique to each agent.^[24–26]^ Numerous immunosuppression protocols are used by transplant centers worldwide, while each LTx recipient might need an individually customized immunosuppression regimen to keep a balance between the benefits and detriments of therapy.^[24–26]^ LTx recipients are maintained on lower levels of immunosuppression compared to other solid organ transplant recipients. Furthermore, long-term allograft survival is achieved in some LTx recipients, even after immunosuppression withdrawal.^[27–29]^ Progresses made within the past 3 decades in describing the mechanisms of immune response have provided multiple therapeutic options to reduce the rejection rates and improve the graft survival in transplantations, as well as providing alternatives to cytotoxic therapy in immune-mediated diseases.^[30,31]^

Post-transplant immunosuppressive regimens are based on a calcineurin inhibitor; either cyclosporine or tacrolimus. These medications were shown to have a similar mechanism of action through inhibition of calcineurin phosphatase.^[32,33]^ Tacrolimus acts as an immunomodulatory agent by inhibiting the transcription of the gene encoding interleukin-2 which is necessary for the T-cell-mediated immune response.^[32,33]^ The distribution of tacrolimus plays a considerable role in its metabolism. The uptake process begins in the stomach and/or proximal small bowel and the complete disintegration (and presumably optimal absorption) occurs in the distal small bowel or the colon.^[12–14]^ Therefore, this can lead to lower clearance and further increased bioavailability with the potential of toxicity.^[12–14]^ Evidence suggests that neurotoxicity, nephrotoxicity, akinetic mutism, new onset diabetes (post-transplant), gastro-intestinal toxicity, hepatotoxicity, and thrombotic microangiopathy could be associated with high levels of tacrolimus after LTx.^[34]^ As an immediate-release formulation, tacrolimus was first administered twice daily (Prograf). Two formulations of tacrolimus have been provided to be administered once daily: a prolonged-release formulation (Advagraf) and more recently an extended-release formulation (Envarsus).^[32,34–36]^ It is necessary to mention that the blood level of different types of tacrolimus should be determined and compared with each other. It is not yet clear which type is preferred. HDTACRO study is the first study to make a comparative assessment of different types of Tacrolimus. Is cannot be denied that the non-randomized selection of participants is considered as a weakness in this study.

In summary, Tacrolimus revolutionized postoperative care after LTx. Various types of this immunosuppressive drug are known, but it is not yet clear which one is preferred in terms of patient adherence and acceptance, the stability of blood level and also the rate of complications of different types of this immunosuppressive medication. The HDTACRO study will be the first study which systematically and prospectively evaluates various oral Tacrolimus-based immunosuppressives.

As a future perspective, if at the end of the trial a difference between the therapy-subgroups would be evident, a randomized control trial will eventually be designed and conducted after submission to the local ethics committee and federal authorities. All diagnostic tests and medications are in accordance with our clinical routine, without any deviation from standard care in transplant patients.

## Author contributions

**Conceptualization:** Arianeb Mehrabi, Markus Mieth.

**Methodology:** Arianeb Mehrabi, Elias Khajeh, Ali Ramouz, Markus Mieth, Sadeq Ali-Hasan-Al-Saegh, Omid Ghamarnejad.

**Project administration:** Arianeb Mehrabi, Markus Mieth, Elias Khajeh.

**Review and editing:** Karl Heinz Weiss, Christian Rupp, Sepehr Abbasi Dezfouli, Anastasia Lemekhova, Melisa Saracevic, Markus W. Büchler, Arianeb Mehrabi.

**Statistical design:** Elias Khajeh, Ali Ramouz, Sadeq Ali-Hasan-Al-Saegh.

**Writing – original draft:** Elias Khajeh, Omid Ghamarnejad, Ali Ramouz, Ali Majlesara, Sadeq Ali-Hasan-Al-Saegh, Parnian Alamdari, Sepehr Abbasi Dezfouli.

All authors read and approved the final manuscript.
